# Synthesis Characterization of Platinum (IV) Complex Curcumin Backboned Polyprodrugs: In Vitro Drug Release Anticancer Activity

**DOI:** 10.3390/polym13010067

**Published:** 2020-12-26

**Authors:** Honglei Zhang, Yanjuan Wu, Xiao Xu, Chen Chen, Xiukun Xue, Ben Xu, Tianduo Li, Zhaowei Chen

**Affiliations:** 1 Shandong Provincial Key Laboratory of Molecular Engineering, School of Chemistry and Chemical Engineering, Qilu University of Technology (Shandong Academy of Sciences), Jinan 250353, China; B17864099398@163.com (H.Z.); Jasonxue@yeah.net (X.X.); xuben2019@126.com (B.X.); ylpt6296@vip.163.com (T.L.); 2 Institute of Food Safety and Environment Monitoring, College of Chemistry, Fuzhou University, Fuzhou 350108, China; 201310023@fzu.edu.cn (X.X.); 201310006@fzu.edu.cn (C.C.)

**Keywords:** nanosized drug delivery system, dual drug, backboned, combination chemotherapy, polyprodrug

## Abstract

The conventional mono-chemotherapy still suffers from unsatisfied potency for cancer therapy due to tumor heterogeneity and the occurrence of drug resistance. Combination chemotherapy based on the nanosized drug delivery systems (nDDSs) has been developed as a promising platform to circumvent the limitations of mono-chemotherapy. In this work, starting from cisplatin and curcumin (Cur), we prepared a dual drug backboned shattering polymeric nDDS for synergistic chemotherapy. By in situ polymerization of the Cur, platinum (IV) complex-based prodrug monomer (DHP), L-lysine diisocyanate (LDI), and then conjugation with a hydrophilic poly (ethylene glycol) monomethyl ether (mPEG) derivative, a backbone-type platinum (IV) and Cur linkage containing mPEG-poly(platinum-co-Cur)-mPEG (PCPt) copolymer was synthesized. Notably, the platinum (IV) (Pt (IV)) and Cur were incorporated into the hydrophobic segment of PCPt with the fixed drugs loading ratio and high drugs loading content. The batch-to-batch variability could be decreased. The resulting prodrug copolymer then self-assembled into nanoparticles (PCPt NPs) with an average diameter around 100 nm, to formulate a synergetic nDDS. Importantly, PCPt NPs could greatly improve the solubility and stability of Cur. In vitro drug release profiles have demonstrated that PCPt NPs were stable in PBS 7.4, rapid burst release was greatly decreased, and the Pt and Cur release could be largely enhanced under reductive conditions due to the complete dissociation of the hydrophobic main chain of PCPt. In vitro cell viability test indicated that PCPt NPs were efficient synergistic chemotherapy units. Moreover, PCPt NPs were synergistic for cisplatin-resistant cell lines A549/DDP cells, and they exhibited excellent reversal ability of tumor resistance to cisplatin. This work provides a promising strategy for the design and synthesis of nDDS for combination chemotherapy.

## 1. Introduction

Cancer remains one of the most severe global public health problems, and it threatens humans’ health and lives seriously. Among the available treatments, chemotherapy is still the most widely adopted therapeutic mode in clinic for various types of cancers. However, the efficiency of conventional mono-chemotherapy is greatly compromised due to insufficient selectivity to the neoplasia, toxicity to healthy tissues, and easily evolved drug resistance [1,2,3]. Unlike mono-chemotherapy, combination chemotherapy using two or more drugs can activate different signaling pathways, maximize therapeutic efficacy, lower the doses of drugs, and overcome drug resistance [4,5]. Despite the excellent advantages, simple drugs co-administration for cancer is still limited by the poor solubility in aqueous solutions and the various pharmacokinetics of different drugs, which have immensely hindered its application and led to therapy failure [6].

The nanosized drug delivery systems (nDDSs) have been extensively exploited for cancer therapy due to many merits, including improved drug solubility, prolonged blood circulation time, reduced side effects, and increased tumor accumulation via enhanced permeability and retention (EPR) effect [7,8,9,10,11,12,13,14,15,16,17]. Among the nanocarriers, polymeric nanoparticles have received increasing attentions for combinatorial cancer therapy [18,19,20,21]. In general, current polymeric co-delivery systems for combination therapy are largely based on the physical co-encapsulation of chemotherapeutic drugs [22,23]. Zhu and coworkers developed polymersomes from lipid-PEG conjugates with folate as tumor-targeting ligand and PCL–PEG–PCL triblock copolymer; then they investigated simultaneous encapsulation and release of DOX and PTX from the polymersomes [22]. In these systems, uncontrollable drugs’ release and premature burst release during circulation can readily lead to serious side effects and yet unsatisfactory therapeutic efficiency. As an alternative paradigm, covalent conjugation of different drugs to the chain ends or side chains of polymers are the other general approaches to construct the polymeric co-delivery systems [24,25,26]. Covalent conjugation systems are inactive and stable during blood circulation, while they could release drugs in response to stimuli, such as acidic pH, redox potential of tumor, and overexpressed enzymes, thus providing better control on drugs release and resulting in weakened side effects [26]. However, these systems had some disadvantages, such as complicated synthesis process, uncertain loading ratio of multiple drugs, and low drugs loading contents and efficiency. Batch-to-batch variations of drugs loading and release behaviors are often observed in these systems, and the variations may be a key bottleneck to the clinical translation [27].

Recently, prodrug backboned polymers, in which drugs or their derivatives acting as components of polymer backbones instead of coupling onto the end or side chain of the polymers, have been put forward to achieve better control over the composition of nDDSs, as well as increased drug-loading content without burst release [19,28,29,30]. For instance, 10-hydroxycamptothecin (HCPT) (including two hydroxyl groups) [31,32], curcumin (including two hydroxyl groups) [28,29], mitoxantrone (including two hydroxyl groups) [33], and diamminedichloro-dihydroxyplatinum (DHP) (including two carboxyl groups) have been utilized in the synthesis and preparation prodrug backboned polymers, through which both high drug loading content and enhanced anticancer efficiency were allowed [34]. Usually, only one chemotherapeutic drug was introduced into the backbone of polymer. Two or more drugs backboned polymeric nanocarriers are still critical and urgent for combination chemotherapy.

**Scheme 1:**
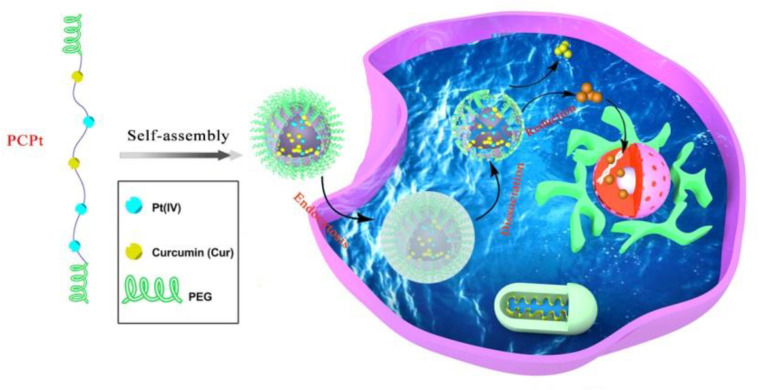
Schematic illustration of the self-assembly and intracellular trafficking pathway of mPEG-poly(platinum-co-Cur)-mPEG (PCPt) nanoparticles (PCPt NPs).

It is known that curcumin (Cur), a promising natural anticancer ingredient, has been brought into research due to its pharmacologically safety and potential to overcome multidrug resistance property [35,36,37]. Unfortunately, application of Cur is limited by its poor water-solubility and instability [29]. Nanotechnology-based strategy was developed for Cur delivery, to overcome associated challenges which restrict Cur therapeutic application. Among various nanoparticle platforms, liposomes have been extensively investigated for curcumin delivery due to the biocompatibility, biodegradability, and safety of liposomes [38,39,40,41]. In general, curcumin was introduced into the phospholipid bilayer via passive loading methods, which have some disadvantages, such as limited capacity, instability, and burst-release profile. On the other hand, cisplatin (cis-dichlorodiammineplatinum(II)) is one of the most effective and widely used therapeutic agents against many cancers in clinic. The oxidation of cisplatin to platinum (IV) (Pt(IV)) compounds is a promising strategy to minimize its severe side effect [42,43]. Previous reports have shown that combinational use of cisplatin and curcumin could synergize cancer therapy [44,45]. With these in mind, herein, Pt(IV)-complex-based prodrug with bis-isocyanate group was first synthesized. Then, this prodrug could be copolymerized with curcumin and modified with hydrophilic methoxypolyethylene glycol (mPEG), to form a dual prodrug backboned polymer mPEG-poly(platinum-co-Cur)-mPEG (denoted as PCPt), which could self-assemble into spherical nanoparticles in aqueous solution (denoted as PCPt NPs) (Scheme 1). Pt(IV) and curcumin units are incorporated in the hydrophobic polyprodrugs’ backbone as a part of the carrier, resulting in high drugs loading contents and efficiency without premature burst release. It is anticipated that PCPt NPs had excellent biostability and cell-imaging performance. After endocytosis by cancer cells, the hydrophobic polymer backbone would be cleaved under reductive microenvironment of the tumor cells, active Pt(II) drug could be released and turn into pharmaceutically active conformations. Meanwhile, curcumin could be released under acid endo/lysosome and enzyme microenvironment, thereby exhibiting synergistic chemotherapy. The stability, drugs-loading capacity, in vitro drugs release profiles, cellular uptake and imaging, and cytotoxicity were further conducted to evaluate the feasibility of this polyprodrugs in detail. The PCPt NPs system possesses many superiorities: (1) Drugs were polymerized to form the hydrophobic segment, high drugs-loading content and loading efficiency could be achieved, and batch-to-batch variability could be decreased. (2) A high drug-to-polymer ratio would reduce the dose required in a given fundamental research. (3) The backbone-type prodrugs PCPt NPs were site-specifically chain-shattered via the reduction of Pt(IV) to Pt(II) in the endosomal/lysosomal reductive intracellular microenvironments, making the Pt and Cur release in a controlled manner, moreover, PCPt NPs had the synergistic inhibitory effects against cisplatin-resistant A549/DDP cells compared to cisplatin and free Cur.

## 2. Materials and Methods 

### 2.1. Materials

Cisplatin was purchased from Shandong Boyuan Pharmaceuti-cal Co., Ltd. (99.8%, Jinan, Shandong, China). Poly(ethylene glycol) monomethyl ether (mPEG_5k_-OH, Mn = 5000 g/mol), 3-(4,5-dimethylthiazol-2-yl)-2,5-diphenyltetrazolium bromide (MTT, 98.0%), and Hoechst 33,258 (≥98.0%) were bought from Sigma-Aldrich. Cur, L-ascorbic acid sodium salt (SA, 99.0%), hexamethylene diisocyanate (HDI, 99.0%), dibutyltin dilaurate (95.0%), L-Lysine diisocyanate (LDI, 95.0%), and hydrogen peroxide solution (H_2_O_2,_ 30. wt% in water) were obtained from Aladdin Chemistry Co. Ltd. (Shanghai, China). Dulbecco’s modified Eagle’s medium (DMEM)/high-glucose medium, phosphate-buffered saline (PBS) and trypsin were purchased from GE Healthcare Life Sciences. Gibco Fetal Bovine Serum (FBS) was bought from Thermo Fisher Scientific Inc. (Shanghai, China). All chemical reagents were of analytical grade, without further purification, if not mentioned otherwise. Dichloromethane (DCM, ≥99.5%, Sinopharm Chemical Reagent Co., Ltd., Shanghai, China), dimethyl sulfoxide (DMSO, ≥99.0%, Sinopharm Chemical Reagent Co.,Ltd., Shanghai, China), and N,N-Dimethylformamide (DMF, ≥99.5%, Sinopharm Chemical Reagent Co.,Ltd., Shanghai, China) were dried with calcium hydride (CaH_2_, 95.0%, Aladdin Chemistry Co. Ltd., Shanghai, China) for 2 weeks before further purification.

### 2.2. Methods

Nuclear magnetic resonance (NMR) spectra were recorded, at room temperature (RT), on a Bruker AVANCE II 400 NMR spectrometer. Gel permeation chromatograph (GPC) measurement was performed on Wyatt Technology’s DAWN (SEC-MALS) instrument to determine the molar masses and polydispersity index of polymers. Fourier Transform Infrared (FTIR) measurements were operated on a Thermo Scientific Nicolet IS10 instrument. UV–visible absorption spectra were determined on a Cary 5000 UV–Vis–NIR spectrometer. Fluorescence spectra were recorded on Hitachi F-4600 spectrometer. The particle size and size distribution were measured by the dynamic light scattering (DLS) using a Malvern Zetasizer Nano ZS90 instrument. Transmission electron microscopy (TEM) images were taken on a JEOL JEM 2100 electron microscope. Platinum content measurements were performed on the inductively coupled plasma mass spectrometry (ICP–MS, Optima 2000DV, PerkinElmer, Waltham, MA, USA). Confocal laser scanning microscope (CLSM) (Leica SP8, Wetzlar, Germany) was used to observe the in vitro cellular fluorescence imaging. Quantitative fluorescent analysis of cellular uptake was measured by a flow cytometry imaging system (BD FACSCalibur, BD Bioscience, San Jose, CA, USA).

### 2.3. Synthesis of Isocyanate Functionalized Poly(Ethylene Glycol) Monomethyl Ether (mPEG_5k_-NCO)

The mPEG_5k_-OH was activated with HDI, to synthesize the NCO-modified mPEG_5k_-OH (mPEG_5k_-NCO). The mPEG_5k_-NCO was prepared according to the previous published paper with slight changes [46]. Briefly, mPEG_5k_-OH (3 g, 0.6 mmol) was azeotropically dried with toluene (100 mL) and then re-dissolved in dried DCM (20 mL). HDI (2.9 mL, 18 mmol) was dissolved with 50 mL of dried DCM. Then, mPEG_5k_-OH solution was added dropwise into HDI, and a few drops of dibutyltin dilaurate were added subsequently. The admixture was refluxed under argon atmosphere for 20 h. After concentration, the crude product was precipitated into 500 mL of petroleum ether. The dissolution and precipitation procedures were repeated two more times. Afterwards, the final product was dried under vacuum and stored at −4 °C, in a sealed vial, under N_2_ atmosphere, for further use.

### 2.4. Synthesis of Cur and Pt(IV)-Backbone Prodrug Polymer [mPEG-poly(platinum-co-Cur)-mPEG (PCPt)]

**Scheme 2:**
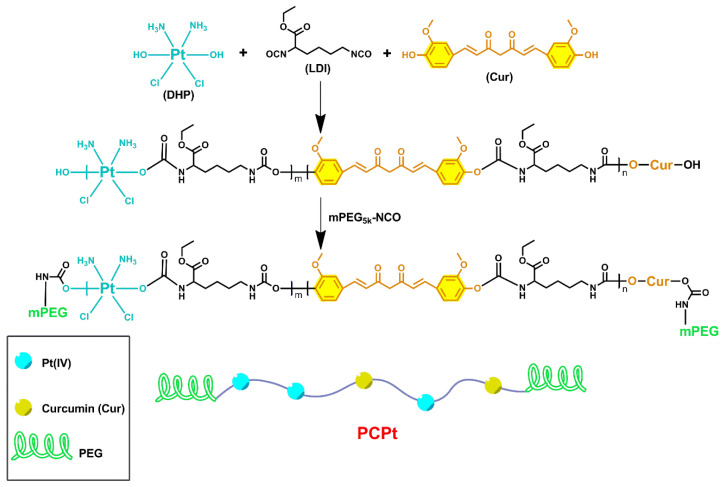
Schematic illustration of the synthesis procedure of PCPt.

Cis, cis, trans-[Pt(NH_3_)_2_Cl_2_(OH)_2_] (DHP) was synthesized according to our previous reported literature [47]. Cur was recrystallized by water/DMF before use. DHP (134 mg, 0.4 mmol) was dispersed in dried DMSO (2 mL). LDI (181 mg, 0.8 mmol) was dissolved in dried DMSO (2 mL) and added dropwise to the above mixture, under stirring. The reaction was allowed to react under argon, at 50 °C, for 8 h. After cooling down to room temperature, Cur (184 mg, 0.5 mmol) in 2 mL of dried DMSO, was added. Then, the reaction mixture was maintained under argon at 50 °C for 24 h. Subsequently, mPEG_5k_-NCO (750 mg, 0.15 mmol) in DMSO solution (10 mL) was added quickly, and the mixture was stirred at 50 °C for another 24 h. The solution was dialyzed against deionized water (MWCO = 14,000) for 72 h, to remove DMSO and unreacted monomers. After lyophilization, the target product, PCPt, was obtained. The molecular weight and polydispersity index of PCPt were determined by GPC with DMF as eluent. The specific synthetic route is shown in Scheme 2.

### 2.5. Preparation and Characterization of PCPt Nanoparticles (PCPt NPs)

The PCPt NPs were prepared as follows: PCPt (20 mg) was dissolved in 2 mL of DMSO, at room temperature, in dark condition. The solution was dropwise injected into 20 mL of Milli-Q water, under stirring. After 4 h, the suspension was dialyzed against deionized water, for 48 h, to remove DMSO. Then, the solution was filtered through a 0.45 μm filter membrane, to obtain PCPt NPs. The mixture was stored at 4 °C for further characterizations and use. The particle size, size distribution, and morphology were characterized by DLS and TEM. The Pt/Cur loading content and Pt/Cur-loading efficiency in the filtrate were calculated according to previous methods [43,48].

Pyrene was used as a fluorescent probe to measure the critical aggregation concentration (CAC) values of PCPt NPs. The pyrene solution was vortexed well with different concentrations of PCPt NPs ranging from 4.9 × 10**^−^**^5^ mg/mL to 0.5 mg/mL, and the solution was kept overnight, to record fluorescence on a fluorescence spectrophotometer with an emission wavelength of 373 nm.

### 2.6. PCPt NPs Stability

The colloidal stability of PCPt NPs under different conditions was determined by DLS and TEM. In detail, lyophilized PCPt power was weighted and resuspended in phosphate-buffered saline (PBS) (0.01 M, pH 7.4), PBS (0.01 M, pH 5.0), MilliQ-water, or MilliQ-water with 10 mM SA. The concentration of PCPt NPs was fixed at 1 mg/mL. At predetermined time intervals, the morphology, size, and size distribution of PCPt NPs were analyzed by DLS and TEM, respectively.

Then, the physiological stability of free Cur and PCPt NPs was studied using UV–vis spectroscopy by characterizing the changes in the absorbance at 425 nm. Briefly, Cur and PCPt NPs were dispersed in PBS buffers of pH 7.4, and incubated in the dark at 37 °C. At a predetermined time point, the sampling was carried out, and the UV–vis absorption spectra of Cur were recorded from 200 to 800 nm.

### 2.7. In Vitro Drug Release Profiles

To evaluate the reduction-triggered drugs release, the release profiles of platinum and Cur from PCPt NPs were measured by using a dialysis method. In general, lyophilized PCPt NPs (5 mg) were hydrated in PBS buffers of pH 7.4 (5 mL), and MilliQ-water (5 mL) with SA (10 mM), respectively. Then, the suspension was introduced into a pre-swelled dialysis bag (MWCO = 3500) and immersed into the aforementioned corresponding solution (35 mL). The dialysis was performed in a shaking culture incubator, at 37 °C, with constant agitation at 100 rpm. At desired time intervals, 2 mL of dialysate was sampled and then fresh corresponding solution (2 mL) was supplemented to the dialysate. Then, 1 mL of sample was used for the determination of Cur concentration. The Cur concentration was calculated by determining the UV–Vis spectra absorbance against its standard calibration curve. A total of 1 mL of residue sample was used for the determination of Pt concentration, and the platinum content was detected via ICP–MS.

### 2.8. Cell Culture

The human cervical carcinoma cell line (HeLa), lung carcinoma A549 cells, and cisplatin-resistant lung carcinoma A549 cells (A549/DDP) were bought from the institute of biochemistry and cell biology, Chinese Academy of Sciences, Shanghai, China. The cells were cultured in complete DMEM medium with 10% heat-inactivated FBS, 100 IU/mL penicillin–streptomycin, and incubated at 37 °C with 5% CO_2_. The DMEM culture medium was re-freshened every 2 days, to maintain the exponential growth.

### 2.9. Cytotoxicity Assay

Cytotoxicity was evaluated by standard MTT assay according to the manufacturer’s protocol. Cisplatin is a broad-spectrum anticancer drug that is widely used in clinic for treating more than 50% of cancers, such as ovarian, lung, prostate, and cervical cancer, etc. Curcumin, a phytochemical, has a wide range of therapeutic activities, including the potent antiproliferative effects against various tumors in vitro. The anticancer effect of curcumin on cervical cancer has been extensively studied. Therefore, HeLa cell lines were chosen as model cells. The tests were given to four groups as follows: cisplatin, Cur, DHP, and PCPt NPs. In brief, each well of 96-well plate was seeded with 5000 cells in 100 μL of DMEM media, and the cells were cultured for 24 h to allow cell attachment. The medium was replenished with fresh DMEM, and then each sample drug with a final Pt/curcumin concentration from 6.25 to 216 µM was co-cultured with cells. After 48 or 72 h of incubation, the medium was changed back to fresh DMEM, and 20 μL of MTT reagent (5 mg/mL in PBS 7.4) was added. The cells were incubated for another 4 h. Afterwards, the culture medium was carefully aspirated, 150 μL of DMSO was used to dissolve the purple MTT-formazan crystals generated by live cells, and the absorbance of the formazan product was measured at 490 nm using a microplate reader. Cells without drug co-incubation were set as controls. 

To evaluate the in vitro efficacy of overcoming multidrug resistance (MDR), cell viability of A549 and A549/DDP against cisplatin, Cur, DHP, and PCPt NPs were measured with a MTT assay. Cells were seeded onto 96-well plates (5000 cells/well). After incubation for 24 h, cells were exposed to the drugs with different concentrations (6.25 to 216 µM). After co-incubation for 48 h, and cells were treated as depicted above. Cells treated with blank DMEM media were negative controls.

Combination index (CI) was calculated with the following equation:

$$CI=\frac{C_{a,x}}{IC_{x,a}}+\frac{C_{b,x}}{IC_{x,b}}$$

*ICx*,*a* and *ICx*,*b* are the concentrations of drug A (DHP) and drug B (Cur) used alone to produce x% cell kill. *Ca*,*x* is the concentration of DHP to achieve same x% effect in combination with Cur, and *Cb*,*x* is the dose of Cur to achieve same x% cell kill in combination with DHP. The CI values lower than, equal to, and higher than 1 indicate synergism, additive, and antagonism effect, respectively.

### 2.10. In Vitro Cellular Uptake

The cellular uptake of free Cur and PCPt NPs was followed with CLSM. HeLa cells were inoculated into a 6-well plate with a density of 2 × 10^5^ cells per well in 2 mL DMEM media and incubated overnight. The solutions (500 μL) of free Cur and PCPt NPs were then added and cultured for 0.5 or 4 h, respectively. The final concentration of Cur was fixed at 2 μg/mL. Prior to characterization with CLSM, cells were rinsed with cold PBS 7.4 for three times to dislodge drugs, fixed with 4% paraformaldehyde (in PBS 7.4) for 20 min, and then the nuclei were dyed with Hoechst 33,258 (10 μg/mL) for another 10 min. Finally, cells were washed with cold PBS 7.4 for three times and recorded by CLSM.

Besides, in vitro cellular uptake of free Cur and PCPts was quantified by flow cytometry. HeLa cells were planted into 6 well plates at 3 × 10^5^ cells per well in 2 mL DMEM media. After 24 h incubation, free Cur and PCPts (final concentration of Cur fixed at 15 μg/mL) dissolved in 500 μL of PBS were added and cultured for another 1 or 4 h. Then, the original medium was removed, and cells were washed with PBS for 3 times. The cells were detached with trypsin, collected by centrifugation. Finally, cells were resuspended in 500 μL of PBS and were studied by flow cytometer.

## 3. Results and Discussions

### 3.1. Synthesis of PCPt

Cisplatin-based chemotherapy combining with a second-line chemotherapeutic drug is usually the standard treatment for a variety of cancers. Cur has been reported to have anti-infection, anti-inflammatory, and anticancer properties. More importantly, previous studies have indicated that Cur could decrease the side effects caused by cisplatin and enhance the anticancer efficiency of cisplatin [44,45,49]. As shown in Scheme 2, Pt(IV) prodrug (DHP) and Cur were copolymerized with LDI, to construct a hydrophobic block, and then terminated with hydrophilic mPEG_5k_-NCO, to obtain amphiphilic triblock PCPt in this study.

**Figure 1:**
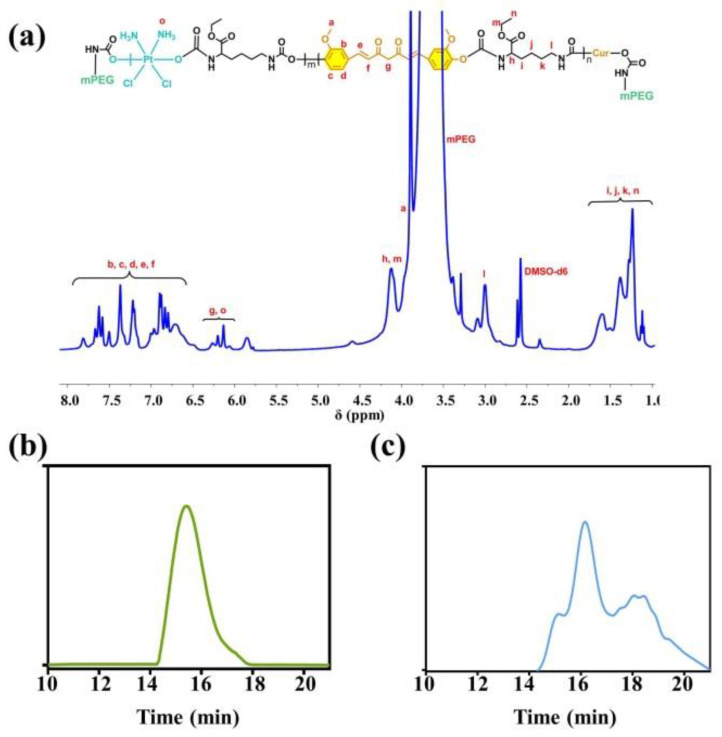
(**a**) ^1^H NMR spectrum of PCPt (400 M, DMSO-d_6_), (**b**) GPC curve of PCPt, and (**c**) GPC curve of PCPt treated with 10 mM SA for 12 h.

The chemical structures of the monomer and copolymer were characterized by ^1^H NMR spectroscopy. The ^1^H NMR results confirmed the preparation of DHP and mPEG_5k_-NCO (Supplementary Materials Figure S1). Figure 1a showed ^1^H NMR spectrum of PCPt copolymer, and all characteristic signals of Cur, Pt(IV), LDI and PEG could be observed clearly. The chemical shift at δ3.49 ppm was attributed to the methylene protons of PEG chain. The peak at δ 2.92 ppm should be attributed to the methylene groups of LDI adjacent to -NH-CO-, and the strong resonance peaks located at δ 1.04–1.73 ppm should be ascribed to methylene and methyl groups of LDI, which confirmed the polymerization of LDI. The peaks at δ 6.63–7.73 ppm were the signals of vinyl protons and benzene protons from Cur segment, and δ 5.98–6.18 ppm was attributed to the protons of -NH_3_ (group of Pt(IV)) and methylene group near the -C=O (group of Cur). Moreover, the polymerization of DHP was illustrated by ICP-MS. In addition, the FT-IR spectra of all monomer and copolymer were also provided in Figure S2. The molecular weight and molecular weight distribution of PCPt was determined to be 17.4 kDa and 1.51 by GPC, respectively (Figure 1b and Table 1). Moreover, GPC was also used to characterize the degradation of PCPt under 10 mM L-ascorbic acid sodium salt (SA) conditions due to the inherent reduction-sensitive property of Pt(IV). As shown in Figure 1c, the GPC curve widened after incubation with 10 mM SA for 12 h, and a series of low-molecular-weight species appeared, which indicated the reduction-activatable dissociation of PCPt. 

### 3.2. Self-Assembly Behaviors

**Figure 2:**
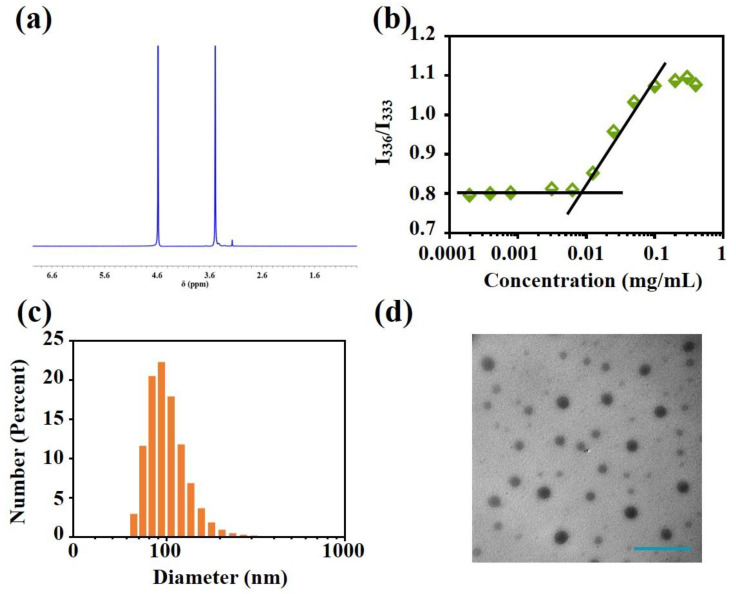
(**a**) ^1^H NMR spectrum of PCPt (400 M, D_2_O), (**b**) intensity ratio (I_336_/I_333_) in the fluorescence spectra of pyrene as a function of PCPt concentrations in aqueous solution, (**c**) particle size distribution of PCPt NPs, and (**d**) TEM morphology of PCPt NPs (scale bar: 500 nm).

PCPt was an amphiphilic ABA triblock polymer, which might be assembled in selective solvents. Firstly, ^1^H NMR spectrum were conducted to investigate the self-assembly behavior. As shown in Figure 2a, compared with ^1^H NMR spectrum in DMSO-d_6_, one signal at 3.51 ppm was assigned to the peaks of the mPEG_5k_ (-OCH_2_CH_2_-), and all the other peaks of PCPt disappeared in D_2_O, which illustrated that PCPt could self-assembled into PCPt NPs in aqueous solution. To further study the self-assembly property, the CAC values of PCPt was measured by using pyrene as fluorescence probe. The CAC of PCPt NPs was determined to be 7.94 × 10^−3^ mg/mL (Figure 2b). Due to the low CAC value, the PCPt NPs are more stable in aqueous solution and capable of long circulation in vivo [50]. Next, the PCPt NPs were prepared via a dialysis method. The average particle size and size distribution of PCPt NPs analyzed by DLS were about 103 nm and 0.19, respectively (Figure 2c and Table 2). The results indicated that the PCPt NPs had a small and uniform size, which would be favorable to prolonged blood circulation and cell endocytosis. As shown in Figure 2d, TEM result revealed that the PCPt NPs were individual spherical particles, which was in line with DLS results. All of these results provided obvious evidence for the process of self-assembly.

### 3.3. Stability Characterization

**Figure 3:**
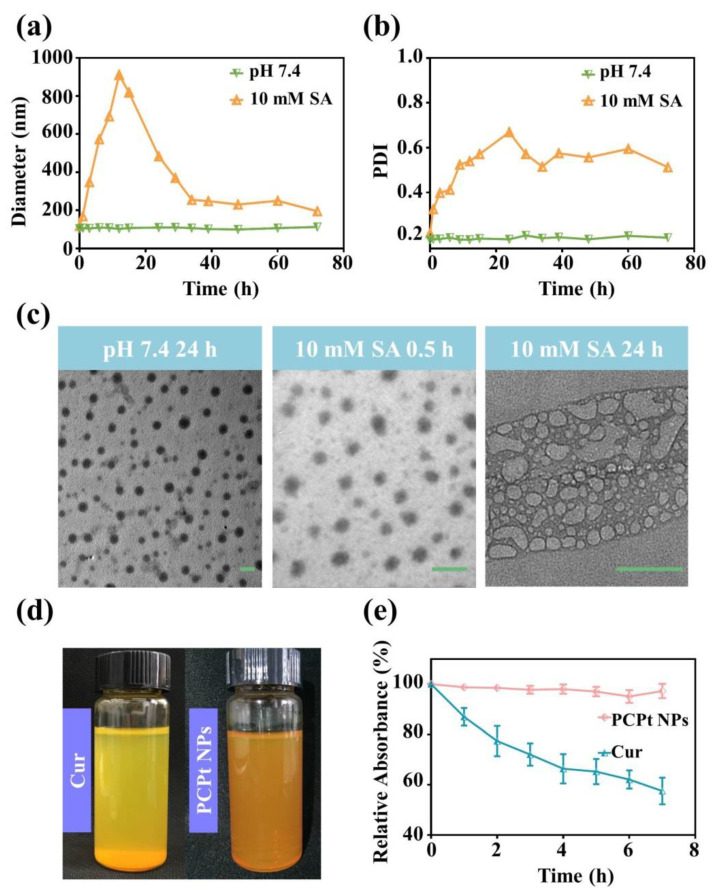
Stability of PCPt NPs and free Cur. (**a**) The hydrodynamic diameter of PCPt NPs measured by DLS at different times (0–72 h). (**b**) PDI of PCPt NPs measured by DLS at different times (0–72 h). (**c**) TEM images observed for the morphology change of PCPt NPs after incubation at different conditions (scale bar: 100 nm). (**d**) Optical photographs taken for PCPt NPs and free Cur in PBS 7.4. (**e**) PCPt NPs and free Cur relative absorbance in PBS 7.4, at different time points, under dark condition.

It was reported that Pt(IV) prodrugs could be reduced into Pt(II) drugs by reductive agents, such as DTT and GSH, but this was not expected for the drug-delivery system during blood circulation. Herein, the particle size and polydispersity index (PDI) of PCPt NPs were characterized in vitro by DLS, under various physiological conditions, for different times (Figure 3a,b and Table 2). According to the DLS results, there is no visible difference in terms of average size and PDI of PCPt NPs in PBS 7.4, even after 72 h incubation. However, in the presence of 10 mM SA, both particle size and PDI of PCPt NPs dramatically changed, the size increased rapidly from 103 to 911 nm within 12 h, and then the size gradually decreased, which could be due to the degradation of backbone. Next, the stimuli responsiveness and corresponding dimensions and morphology changes were further investigated by TEM. In Figure 3c, spherical nanoparticles with an average diameter of ~108 nm could be clearly observed for PCPt NPs incubated in PBS 7.4 for 24 h. In contrast, after incubation with 10 mM SA for 24 h, the morphology and distribution changed greatly, which further confirmed the reduction-sensitive property of PCPt NPs.

Free Cur is a hydrophobic anticancer drug, and its saturated concentration in water is determined to be around 0.6 μg/mL [48]. The nanosized curcumin can exhibit increased solubility and stability [48,51]. As displayed in Figure 3d, the nanosized PCPt NPs solution was homogeneous and could maintained one month with no precipitation, while free curcumin solution was heterogeneous, and visible precipitate was clearly observed after standing for 10 min. The partition coefficient (logP) of curcumin, DHP, and PCPt NPs between n-octanol and water was determined by the traditional shake flask method, at 25 °C, with the assistance of HPLC (Supplementary Materials Figures S4 and S5). The logP of Cur, DHP, and PCPt NPs was determined to be 0.746, −0.418, and −0.682, respectively. Conjugated Cur in the form of PCPt NPs showed enhanced water solubility and stability relative to free Cur. More importantly, the physiological stability of nanoparticles is a vital concern in drug delivery. To excess the physiological stability of PCPt NPs and free Cur in PBS (pH 7.4), the change in their absorbance was monitored by UV–vis spectroscopy. The UV–Vis spectrum and fluorescence spectrum of PCPt NPs are displayed in Supplementary Materials Figure S3. Absorbance of Cur at around λmax = 425 nm was used to appraise the chemical stability of it. As shown in Figure 3e and Supplementary Materials Figure S6, the degradation rate of free Cur in PBS 7.4 was really rapid and the Cur content remained only 59.9% after 7 h incubation under dark. Compared to in PBS 7.4, the degradation rate of free Cur in PBS 5.0 was slower (Supplementary Materials Figure S7). The result was in accordance with the previous reports [51,52]. In contrast, the absorbance change of PCPt NPs was much slower than the free Cur, more than 94% of Cur in the PCPt NPs kept normal after 7 h in PBS 7.4. These results demonstrated that the conjugation and formation of nanoparticles could protect free Cur from degradation, and the stability of Cur was significantly improved.

### 3.4. Pt and Cur Release Profile

**Figure 4:**
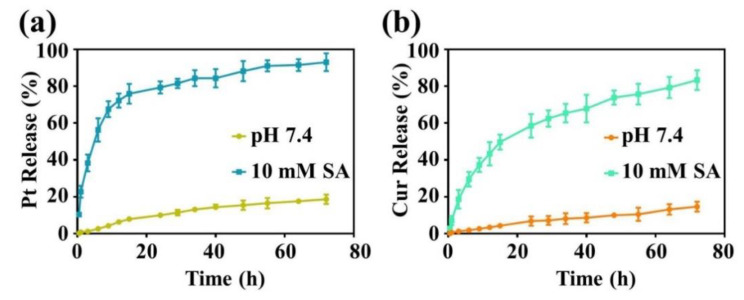
(**a**) Release profiles of Pt from the PCPt NPs under different conditions. (**b**) Release profiles of Cur from the PCPt NPs under different conditions.

In order to achieve combination chemotherapy, cisplatin derivative was copolymerized with curcumin to give a co-delivery system (PCPt NPs). The ideal nanodrugs for chemotherapy should possess several merits. One of the merits is high drug loading content, high drug loading efficiency and generation of sufficient concentration in the target cancer cells. Thus, the drugs concentration is important. The drug loading content (DLC) of Pt and Cur were calculated to be approximately 9.7% and 9.1%, respectively. Since Pt(IV) was conjugated onto the backbone of PCPt, drug release behaviors of the PCPt NPs would be reduction dependent. The release of Pt and Cur from PCPt NPs was investigated using a dialysis method in PBS (pH 7.4) or 10 mM SA. As shown in Figure 4a, rather slow Pt release was detected at pH 7.4, only about 18.8% of the Pt was released after 72 h, indicating that the PCPt polymer and NPs are stable in neutral condition. As expected, the release rate of Pt was increased significantly upon the addition of 10 mM SA, the cumulative Pt release amount reached to 93.3% after 72 h, which proved that the Pt release was reduction-dependent, and PCPt NPs could be degraded at reductive environment based on the breakage of Pt(IV). The results of Cur release were similar to Pt release (Figure 4b), the release of Cur from PCPt NPs was significantly enhanced under reduction condition (10 mM SA). PCPt NPs showed approximately 83.3% Cur release after 72 h incubation with 10 mM SA, only 14% of Cur was released at pH 7.4. Notably, the in vitro release of Cur was slightly lower than Pt in aqueous solution with 10 mM SA, which might be due to the lipophilicity of Cur. Therefore, the reduction responsive PCPt NPs held great potential in developing smart carriers for the combination treatment of cancer. Pt and Cur was introduced into the PCPt via urethane linkage. The urethane linkage could be slowly hydrolyzed, and the conjugated drugs were released. Therefore, the Pt and Cur release in the PBS 5.0 was also investigated (Supplementary Materials Figure S8). Compared to the drugs released in PBS 7.4, the release rate was slightly increased.

### 3.5. In Vitro Cytotoxicity and Cellular Uptake

**Figure 5:**
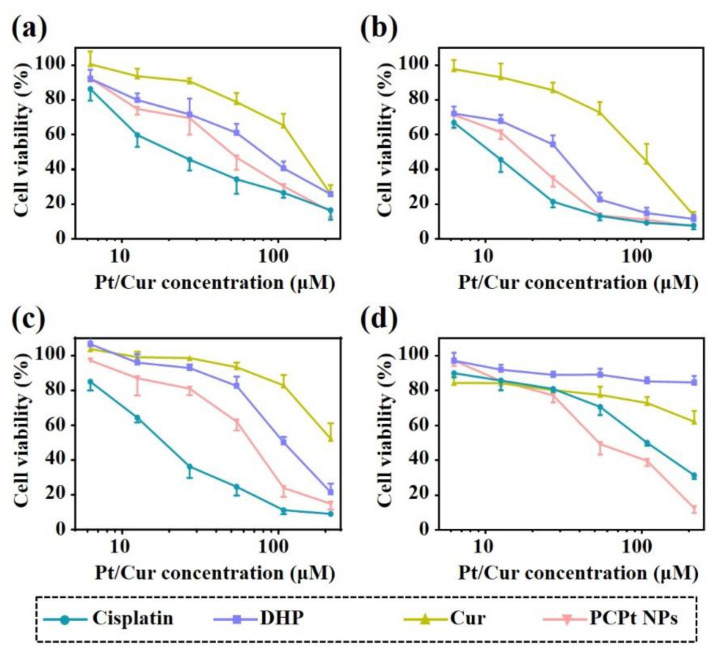
(**a**) The viability of HeLa cells incubated with various drugs and PCPt NPs at different concentrations for 48 h. (**b**) The viability of HeLa cells incubated with various drugs and PCPt NPs at different concentrations for 72 h. (**c**) The viability of A549 incubated with various drugs and PCPt NPs at different concentrations for 48 h. (**d**) The viability of A549/DDP cells incubated with various drugs and PCPt NPs at different concentrations for 48 h. The error bars are based on the standard deviations of four parallel samples.

Cisplatin is widely used for the treatment of various solid malignancies in clinic. However, the major impediments for successful application of cisplatin are the serious side effects on normal cells and inability to target cancer cells. Moreover, the most desirable effects of nDDS for combination cancer therapy are improved biosafety and enhanced cytotoxicity compared with mono-drug therapy. Then, the cytotoxicity of cisplatin, DHP, Cur, and PCPt NPs against tumor cell lines Hela cells was evaluated by MTT assay. As shown in Figure 5, all the drugs induced cytotoxicity in HeLa cells in a dose- and time-dependent manner. The cell viability was gradually decreased with concentration increased and incubation time extended. The viability values demonstrated in Figure 5a indicated that cisplatin showed the highest cytotoxicity to HeLa cell lines after 48 h of incubation; the half-maximal inhibitory concentration (IC_50_) value was about 22.5 μM. Free Cur exhibited greatest cell viability at same concentrations. Moreover, compared with cisplatin, the cell viability of HeLa cells cultured with DHP was higher in the concentration range of 6.25–216 μM, which indicated that Pt(IV) had improved safety. Under the same conditions, the anticancer activity of PCPt NPs was lower than cisplatin. The reason is that the reduction of Pt(IV) prodrug to active Pt(II) need more time, and also might be due to the relatively slow release of drugs. Importantly, PCPt NPs showed enhanced cytotoxicity toward HeLa Cells in comparison with DHP and Cur. Specially, for 50% cell inhibition, the combination index (CI) values were calculated to 0.74 for HeLa cells, which confirm the synergy of platinum and Cur in PCPt NPs. For 72 h incubation, similar trends were observed (Figure 5b). Cytotoxicity of cisplatin, Cur, DHP, and PCPt NPs against A549 and cisplatin-resistant A549/DDP cells at various concentrations were further evaluated by MTT assay. As shown in Figure 5c,d and Table 3, A549/DDP cells had high drug resistance to ciaplatin compared to A549 cells. The new PCPt NPs also were more cytotoxic than ciaplatin and curcumin to A549/DDP cells. IC50 of PCPt NPs in A549/DDP cells was greatly lower than that of cisplatin. Thus, PCPt NPs had the greatest inhibitory effects against A549/DDP cells compared to cisplatin and curcumin. This enhanced inhibitory effect could be attributed to the overcoming MDR by curcumin.

**Figure 6:**
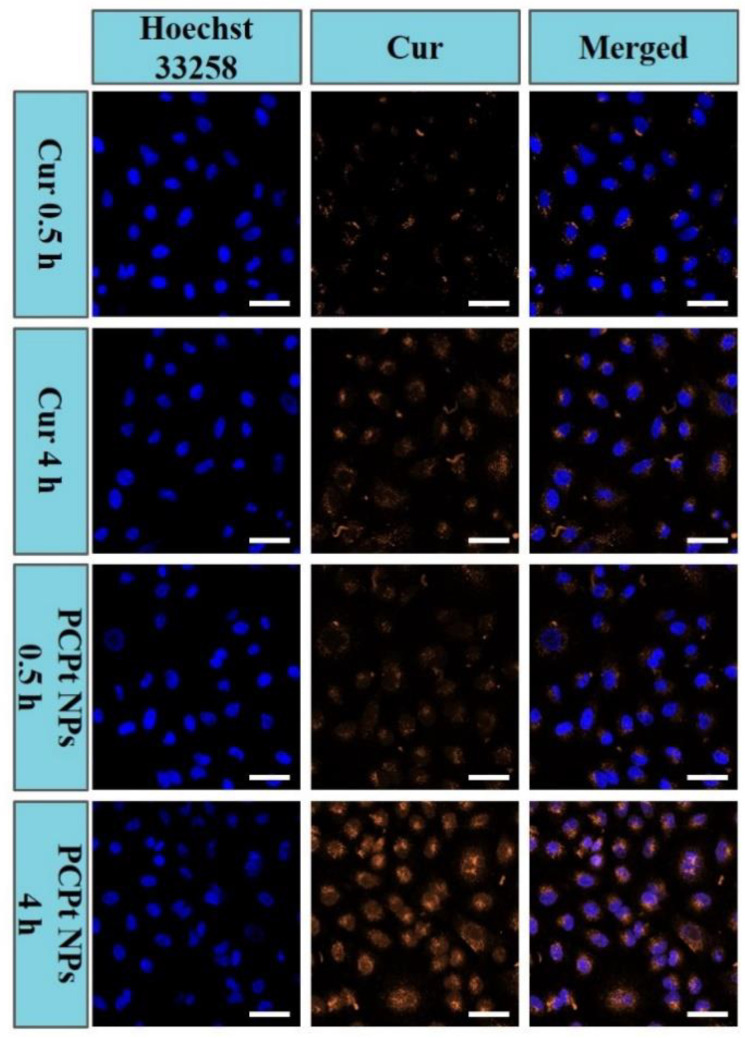
Cellular uptake and intracellular distribution of Cur in HeLa cells observed by using CLSM after 0.5 and 4 h incubation of free Cur and PCPt NPs (Scale bar: 50 μm).

Taking advantage of the intrinsic fluorescence property of Cur molecule, cellular uptake and intracellular distribution of the free Cur and PCPt NPs in HeLa cells were imaged by using CLSM. As illustrated in Figure 6, it could be seen clearly that only a weak yellow fluorescence (Cur) signal was detected mainly in the cytoplasm of the cells after co-incubated with the free Cur for 0.5 h, and similar results were found in PCPt NPs co-incubated HeLa cells. The intracellular uptake of free Cur and PCPt NPs exhibited a time-dependence property. The uptake of free Cur was enhanced, following a prolonged incubation time of 4 h, and the fluorescence was observed not only in the nucleus but the cytoplasm of the cells. Notably, for HeLa cells incubated with PCPt NPs, the fluorescence density was markedly augmented from 0.5 h to 4 h, and the Cur fluorescence was mostly located in the perinuclei and nuclei region of cells. In other words, PCPt NPs gradually released Cur to cytoplasm and then diffused to nuclei. The different intracellular distribution of Cur might be due to the reduction of Pt(IV) to Pt(II) of PCPt NPs in endosome. These imaging results indicated that, compared to the poor water-soluble and stability of Cur, PCPt NPs could be easily internalized by cancer cells and released drugs in a reductive environment.

**Figure 7:**
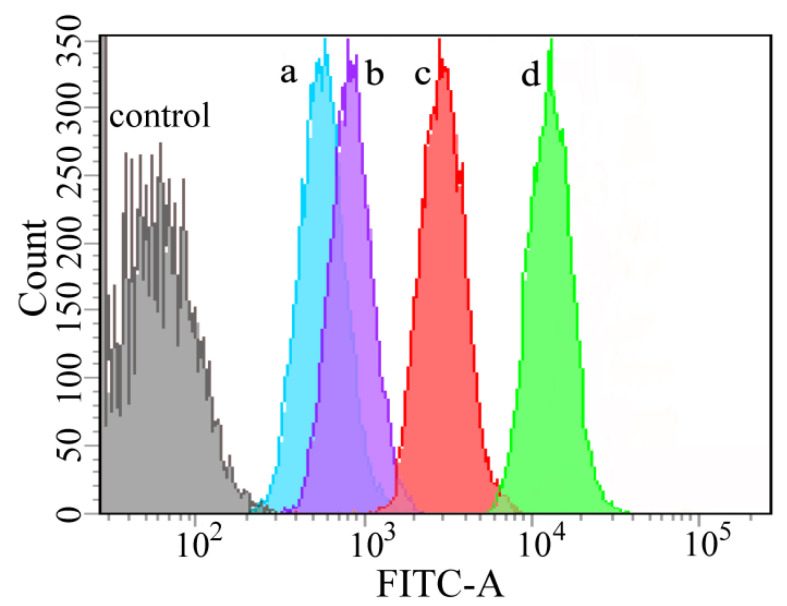
Flow cytometry analysis of HeLa cells treated with free Cur for 1 h (**a**), PCPt NPs for 1 h (**b**), free Cur for 4 h (**c**), and PCPt NPs for 4 h (**d**).

Accordingly, the quantitative analyses of the cellular uptake against HeLa cells were determined by flow cytometry (Figure 7). The PCPt NPs presented a stronger fluorescence level than free Cur after incubation for 4 h. The results were consistent with CLSM results, and they further demonstrated that the PCPt NPs could be rapidly internalized and release the drugs into cytoplasm.

## 4. Conclusions

In summary, a backbone-type platinum (IV) and Cur containing mPEG-poly(platinum-co-Cur)-mPEG (PCPt) copolymer was designed and synthesized by using Cur and platinum (IV) prodrugs DHP as co-monomers. Due to its amphiphilic structure, PCPt could self-assemble into nanoparticles in aqueous solution, which had fixed Pt/Cur ratio and high drug-loading contents. Due to the reduction-responsive property of Pt(IV), the obtained dual-drug-backboned PCPt NPs could respond to the reduction condition in a chain-shattering manner, and released the bio-active Pt(II) and Cur, simultaneously. In vitro cellular assay demonstrated that PCPt NPs exhibited effective intracellular uptake and enhanced cell proliferation inhibition toward the HeLa cells. PCPt NPs has appeared as a promising platform for platinum and Cur co-based combination chemotherapy.

## Figures and Tables

**Table 1:** GPC analysis of mPEG-poly(platinum-co-Cur)-mPEG (PCPt).

Polymer	Number-Average Molecular Weight (kDa)	Molecular Weight Distribution
PCPt	17.4	1.51

**Table 2:** Nanoparticle size (average diameter) and polydispersity index (PDI) determined by dynamic light scattering (DLS).

Nanoparticles (NP)	Average Diameter (nm)	PDI
PCPt NPs incubated in PBS 7.4 for 0 h	103	0.19
PCPt NPs incubated in PBS 7.4 for 72 h	112	0.20
PCPt NPs incubated in PBS 5.0 for 0 h	127	0.22
PCPt NPs incubated in PBS 5.0 for 72 h	297	034
PCPt NPs incubated with 10 mM SA for 0 h	118	0.22
PCPt NPs incubated with 10 mM SA for 72 h	195	0.53

**Table 3:** The IC_50_ of drugs towards different cell lines for 48 h incubation.

Cell Line	Cisplatin	DHP	Cur	PCPt NPs
	IC_50_ of (μM)	IC_50_ of (μM)	IC_50_ of (μM)	IC_50_ of (μM)
HeLa	27.1	54.1	108.2	53.9
A549	26.7	84.8	125.1	54.2
A549/DDP	>216	>216	>216	55.7

## Data Availability

Please refer to suggested Data Availability Statements in section “MDPI Research Data Policies” at https://www.mdpi.com/ethics.

## Supplementary Materials

The following are available online at https://www.mdpi.com/2073-4360/13/1/67/s1. Figure S1: ^1^H NMR spectra of DHP and mPEG_5k_-NCO. Figure S2: Fourier-transform infrared spectra (FTIR) spectra of the DHP, mPEG_5k_-NCO, and PCPt. Figure S3: (a) UV–visible spectrum of PCPt NPs in aqueous solution. (b) Fluorescence spectrum of PCPt NPs in aqueous solution. Figure S6: (a) Variations of fluorescence spectra of free Cur as a function of time. (b) Variations of fluorescence spectra of PCPt NPs as a function of time.
