# Supplementary material for: Synthesis Characterization of Platinum (IV) Complex Curcumin Backboned Polyprodrugs: In Vitro Drug Release Anticancer Activity
Source: Polymers (Basel). 2020 Dec 26;13(1):67. doi: 10.3390/polym13010067 (PMC7795977; doi:10.3390/polym13010067)
Supplement: Supplementary file 1 [file polymers-13-00067-s001.pdf]

Supporting Information

Synthesis and Characterization of Platinum (IV) Complex and Curcumin Backboned Polyprodrugs: in vitro Drug Release and Anticancer Activity

Honglei Zhang [1], Yanjuan Wu[1,*], Xiao Xu [2], Chen Chen [2], Xiukun Xue [1], Ben Xu[1], Tianduo Li [1] and Zhaowei Chen [2,*],

[1] Shandong Provincial Key Laboratory of Molecular Engineering, School of Chemistry and Chemical Engineering, Qilu University of Technology (Shandong Academy of Sciences), Jinan 250353, China;

[2] Institute of Food Safety and Environment Monitoring, College of Chemistry, Fuzhou University, Fuzhou 350108, China

* Corresponding author. E-mail address: wuyanjuan5@163.com; chenzw@fzu.edu.cn;

HPLC equipment and conditions: A Prominence LC-20A (Shimadzu, Japan) equipped with a prominence SPD-20A/20AV UV–Vis detector was used for the HPLC analysis. The samples were separated using a C18 column (200 × 4.6 mm; particle size, 2 μm; Hanbon SCi.&Tech., Jiangsu, China), with mobile phase at a ratio of methanol : 4% acetic acid = 75/25, and the flow rate was 1 mL/min. The column temperature was maintained at room temperature, and the detector wavelength was set and operated at 430 nm. Then, 20 μL of samples were introduced into the HPLC system every 10 min.

Curcumin standard calibration curve: Curcumin samples with different concentrations were introduced into HPLC. The peak area of Cur was plotted against the corresponding concentration to obtain the calibration graph of Cur. The equation of Cur determined by HPLC was as follow: Y=125.9X-30.05 (Y: Peak area; X: Cur concentration)

Solubility analysis: The saturated cur water solution was prepared. After filtration through 0.45 μm hydrophilic nylon membrane filter, the Cur solution was injected into the HPLC system. The concentration of cur was calculated with the assistance of its standard calibration curve. The solubility of Cur in water was about 0.67 μg/mL. The saturated DHP (Pt (IV)) water solution was prepared. After filtration through 0.45 μm hydrophilic nylon

membrane filter, the concentration of Pt was determined by ICP-MS. The solubility of DHP in water was about 86.4 μg/mL. For PCPt NPs, it could be dispersed in the water to form well-distributed and stable mixture up to 2 mg/mL. Notably, PCPt NPs were dispersed rather than dissolved.

Lipophilicity analysis: The partition coefficient of curcumin, DHP and PCPt NPs between n-octanol/water were determined by the traditional shake flask method at 25 °C according to the literature (*Chromatographia*, 2019, 82,809–818). Briefly, curcumin was completely dissolved in the PBS 5.0 at the concentration of 4×10^{-3} mg/mL. 50 mL of the prepared mixture solution and 5 mL of n-octanol were placed in glass-stoppered flasks, and maintained under mechanical agitation for 24 h at 25 °C. The aqueous phases were carefully separated, and measured by HPLC. The partition coefficient (logP) was taken as the logarithm of the ratio between the Cur concentrations measured in the lipophilic and aqueous phases. The logP of Cur was determined to 0.746. The lipophilicity of DHP and PCPt NPs were determined similarly, ICP-MS was used to measure the Pt concentration of aqueous phases. The logP of DHP and PCPt NPs were determined to -0.418 and -0.682, respectively. All the results indicated that the solubility of Cur and DHP could be significantly increased via the PCPt NPs.

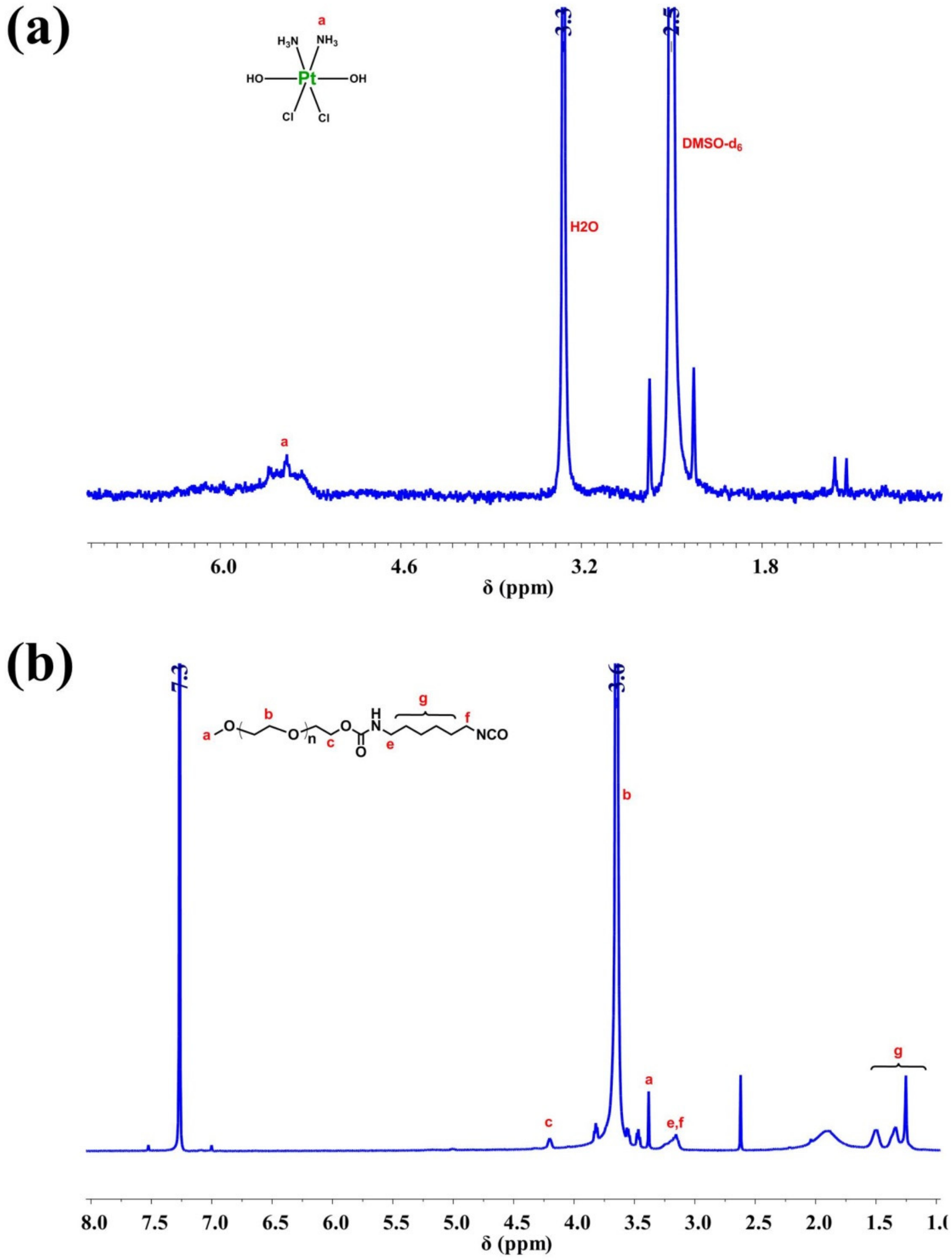

Figure S1. ^1H NMR spectra of DHP (DMSO-d_6), and mPEG$_{5k}$-NCO ($CDCl_3$).

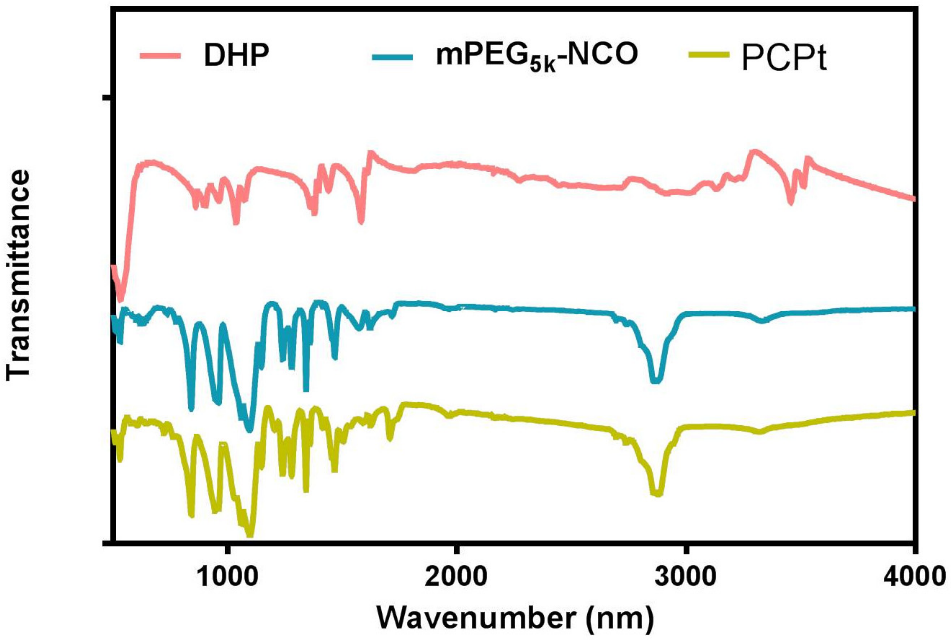

Figure S2. Fourier-transform infrared spectra (FT-IR) spectra of the DHP, mPEG$_{5k}$-NCO and PCPt.

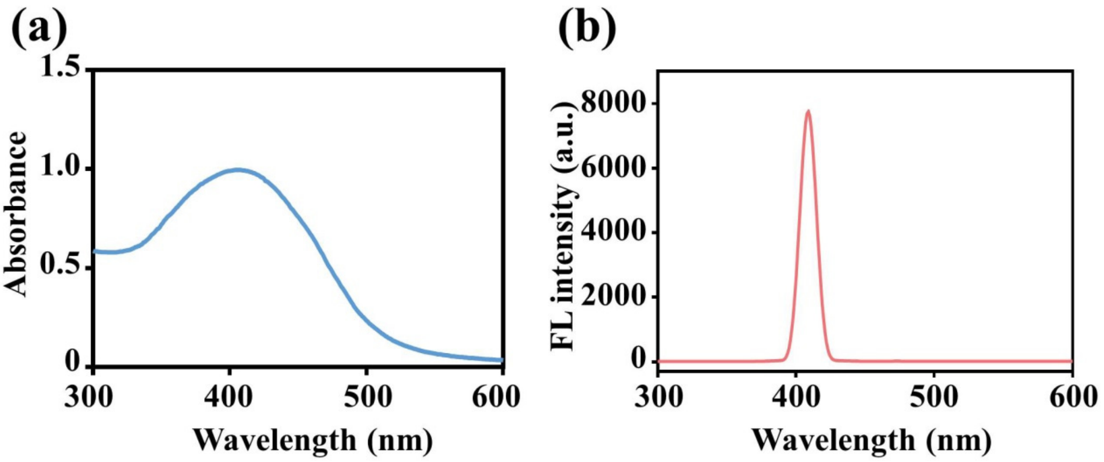

Figure S3. (a) UV–visible spectrum of PCPt NPs in aqueous solution; (b) Fluorescence spectrum of PCPt NPs in aqueous solution.

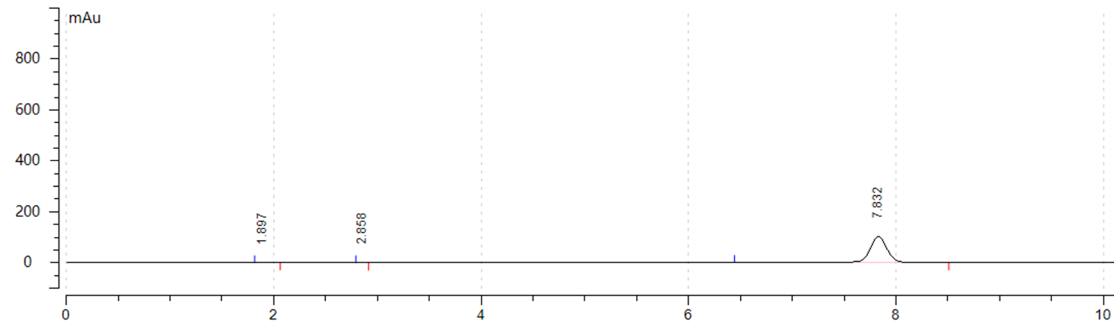

Figure S4. The representative HPLC spectrum of Cur.

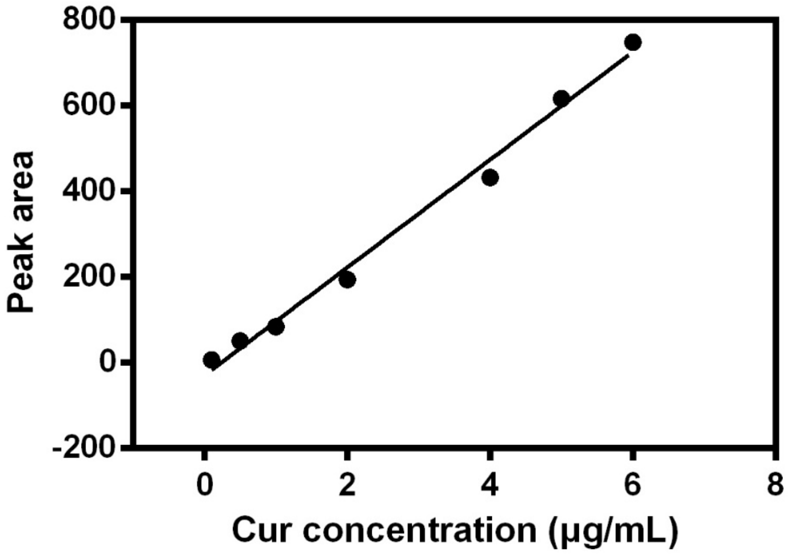

Figure S5. The standard calibration curve of Cur.

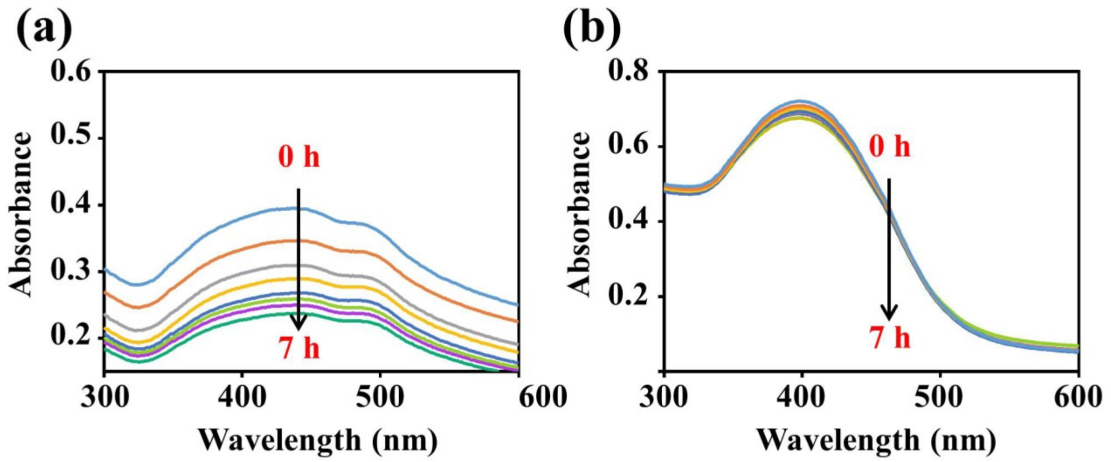

Figure S6. (a) Variations of fluorescence spectra of free Cur as a function of time in PBS 7.4. (b)Variations of fluorescence spectra of PCPt NPs as a

function of time in PBS 7.4.

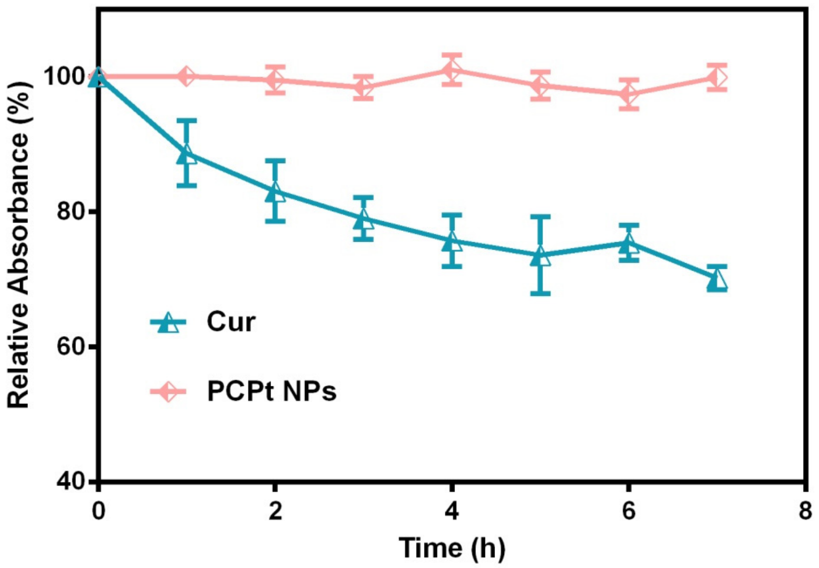

Figure S7. PCPt NPs and free Cur relative absorbance in PBS 5.0 at different time point under dark condition.

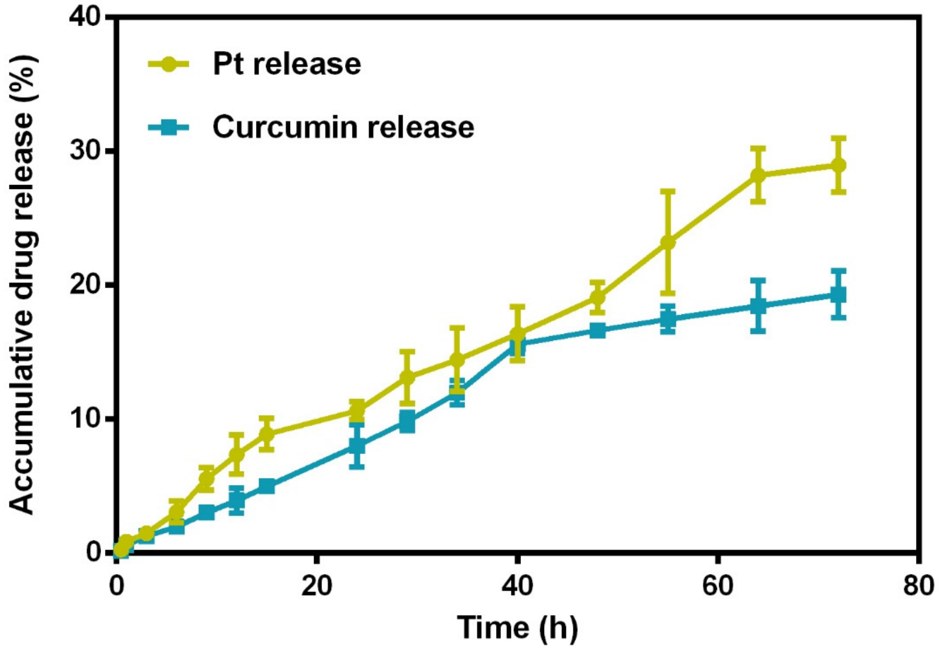

Figure S8. Release profiles of Pt and Cur from the PCPt NPs in PBS 5.0.
